# Virtual screening and evaluation of Ketol-Acid Reducto-Isomerase (KARI) as a putative drug target for Aspergillosis

**DOI:** 10.1186/1559-0275-9-1

**Published:** 2012-02-03

**Authors:** Vivek K Morya, Shalini Kumari, Eun-ki Kim

**Affiliations:** 1 Department of Biological Engineering, Inha University, Incheon, Republic of Korea, 402-751; 2 Department of Biotechnology, H.I.M.T, Greater Noida, U. P., India, 201306

**Keywords:** Aspergillosis, *Aspergillus*, Ketol acid reductoisomerase (KARI), Pharmacophore

## Abstract

Aspergillus is a leading causative agent for fungal morbidity and mortality in immuno-compromised patients. To identify a putative target to design or identify new antifungal drug, against Aspergillus is required. In our previous work, we have analyzed the various biochemical pathways, and we found Ketol Acid Reducto-Isomerase (KARI) an enzyme involves in the amino acid biosynthesis, could be a better target. This enzyme was found to be unique by comparing to host proteome through BLASTp analysis. A homology based model of KARI was generated by Swiss model server. The generated model had been validated by PROCHECK and WHAT IF programs. The Zinc library was generated within the limitation of the Lipinski rule of five, for docking study. Based on the dock-score six molecules have been studied for ADME/TOX analysis and subjected for pharmacophore model generation. The Zinc ID of the potential inhibitors is ZINC00720614, ZINC01068126, ZINC0923, ZINC02090678, ZINC00663057 and ZINC02284065 and found to be pharmacologically active agonist and antagonist of KARI. This study is an attempt to Insilco evaluation of the KARI as a drug target and the screened inhibitors could help in the development of the better drug against Aspergillus.

## Introduction

Various reports from the past two decades point to the occurrence of invasive fungal infections have been greater than ever. *Aspergillus *represents a huge genus of economically, as well as ecologically, important fungi in industry and many fields of applied and clinical research. *Aspergilli *are also a leading cause of fungal morbidity and mortality in immune compromised patients [1-6]. Clinically accessible antifungal agents have quite a few downsides such as restricted potency and spectrum, non-optimal pharmacokinetics, severe resistance and drug-related toxicity. There is an emergent need to develop new antifungal drugs with a new chemical composition and novel mechanism of action [7]. Active efforts are being made by several international agencies and pharmaceutical majors to identify the drug targets and develop new drugs to treat these diseases effectively. To identify an antifungal drug targets for Aspergilli is required to develop new pharmaceuticals, to meet the challenge. Metabolic variations among organisms may be oppressive for the targets for pathogen such as Aspergilli. Because of the huge similarity among Metabolism and enzymes with host, Eukaryotic pathogens such as Aspergilli are always being tedious to control. The information about pathogen and host and their interaction are recurring deposited. A huge database for metabolome, proteome and genome are available, which may exploit for targeting some enzyme, which could be a server for drug designing [7,8]. The KARI has been considered as a target for this study as a result of comparative pathway analysis between host and parasite [8]. This enzyme is involve in biosynthesis of branched chain amino acid (Valine, leucine, isoleucine), Pantothenate and CoA in *Aspergillus*. KARI catalyzes the conversion (s)-2 Aceto-2 hydroxybutanoate to (R)-3-hydroxy 3- methyl 2-oxopentanoate and again KARI utilizes this substrate and produces (R) 2,3-dihydroxy-3-methylpentanoate and converted it into Lucine and Isolucine [8,9]. Parallel to the above, Valine (3-hydroxy 3- methy-l,2-oxobutanoate to 2,3-dihydroxy-3-methylbutanoate) is also synthesized by same pathway. In both the reactions threonine moiety is metabolized into isolucine and valine biosynthesis in *Aspergillus *[10]. For the reaction catalyzed by KARI, Mg^++ ^and NADPH are required as cofactor and coenzyme respectively [11,12]. The KARI and Dihydroxy acid dehydratase are essential enzymes for biosynthesis of Lucine, Isolucine, and Valine and can be targeted as antifungal drug target. Disruption of Lucine, Isolucine and Valine biosynthetic pathway may affect the survival of the *Aspergilli *under the conditions of threonine limitation [8]. Thus, the KARI have selected for this study as as putative Antifungal target. In this present article we have modeled the Aspergillus KARI enzyme, using rice KARI as a template. The modeled structure was validated and used for docking study to find out drug like molecules. The identified molecules were subjected for ADME/T analysis and pharmacophore generation.

## Materials and methods

The criteria for selection of Ketol acid reductoisomerase (KARI) as a drug target have reported in our last manuscript [8]. The sequences of KARI were retrieved from NCBI database http://www.blast.ncbi.nlm.nih.gov.

### Homology modeling

The protein sequence was also obtained from KEGG data base http://www.genome.jp/kegg[13] and the sequence of model of KARI was obtained from NCBI database http://www.blast.ncbi.nlm.nih.gov[14]. Ketol acid reductoisomerase (KARI) enzyme of *Aspergilli *was subjected for homology modeling using Swiss model [14,15]. While possible active site were determined using LIGSITE^csc ^and CASTp web servers simultaneously [16-18]. The structural homologue, which was used as a template for this model, is ketol acid reductoisomerase enzymes from rice with PDB identifier 3fr8B [19]. The sequence similarity between the template and the model is about 33%. The quality of the model was verified using PROCHECK and WHAT IF [20,21] a protein structure verification program. A sequence alignment of Ketol acid reductoisomerase from Rice chain -B and Aspergillus was constructed using the multiple sequence alignment program ClustalX [22].

### Docking

The chemical structures of antagonists for enzyme Ketol acid reductoisomerase were extracted from ZINC. In an effort to make virtual screening more accessible to a large community, it is a free database of purchasable molecules, many of them "drug-like" or "lead-like", in 3D formats compatible with popular docking programs [22]. The ligand molecule was searched on drug databank by submitting the sequence of the enzyme [22,23]. On the basis of information obtained from drug bank, http://www.drugbank.com Library for the antagonist of Ketol acid reductoisomerase were downloaded from the Zinc server within limitation of Lipinski rule's of five [24]. The library retrieved from Zinc http://www.zinc.org was used for Docking.

The docking was performed using Molegro Virtual Docker (MVD), an evaluation version. Molegro virtual docker uses a three-dimensional structure of both protein and ligand (usually derived from X-ray/NMR experiments or homology modeling). MVD performs flexible ligand docking, so the optimal geometry of the ligand will be determined during the docking. Molegro virtual dockers explore the full range of ligand conformational flexibility with partial flexibility of the protein. Docking procedure consisted of three interrelated components; a) identification of binding site b) a search algorithm to effectively sample the search space (the set of possible ligand positions and conformations on the protein surface) and c) a scoring function or energy calculation software [25].

### Pharmacophore mapping

Pharmacophore are the lead compound against a desired target. A pharmacophore is a 3 D arrangement of functional groups within a molecule and these are necessary to bind to a macromolecule or active site Identification of the pharmacophore is an important step in understanding the interactions between receptor and ligand. This was generated with Ligandscout software [26-28]. Pharmacophore of six ligands were generated by this software and align to find out the active site of all [29].

### ADME/T analysis

Pharmacokinetics a term used in the pharmacology which gives idea about Absorption, Distribution, Metabolism and Excretion/Toxicity (ADME/T) of a drug molecule. It has found that more than 50% drugs are fail during clinical trial due to their weak ADME properties [30,31]. Recent advancements in Genomics, Proteomics, High-Throughput Screening (HTS) and the overall drug discovery process have rapidly generated large numbers of potential pharmacologically active compounds waiting for optimization and pre-clinical ADMET evaluation. Thus before clinical trail ADME and toxicity property must be tested. For this analysis we have used Pharma-algorithm server http://pharma-algorithms.com/webboxes/[32].

## Results and discussion

A previous study done in this laboratory about drug target identification through metabolic pathway analysis, total 40 enzymes were found to be essential for *Aspergillus *[8]. When amino acid sequence of KARI was compared with human proteome by BLASTp search, this enzyme was found to be non-homologous. Therefore we have targeted KARI (1.1.1.86) as putative drug target. Some other reasons which make it more interesting is its involvement biosynthesis of lucine, vsoucine and valine and these amino acids are essential for humans. Thus targeting this enzyme will not alter the amino acid metabolism in human while unavailability of these amino acids in pathogen inhibits various pathways.

**Figure 1:**
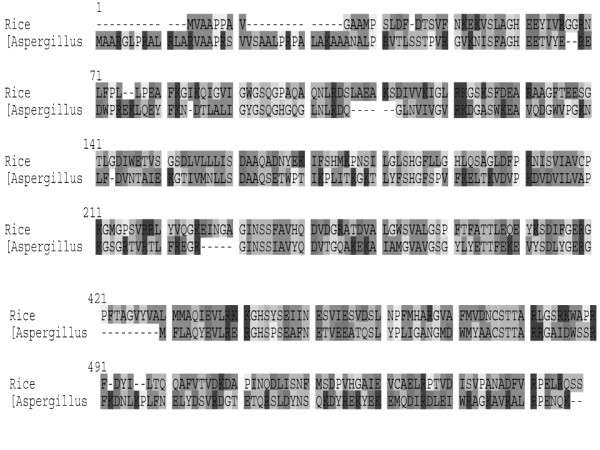
**Sequence alignment of Ketol acid reductoisomerase of Aspergillus with Ketol acid reductoisomerase of Oryza sativa**.

Homology based model of KARI was accomplished by swiss model server [17,18] and the structural homologue, which was used as a template for this model, is ketol acid reductoisomerase enzymes from rice, The PDB identifier 3fr8B [16-18] with a resolution of 2.8 Å. The modeled structure was validated by UCLA server. The exact sequence similarity id about 32.19% in respect to template, therefore the sequence homology between template and subjected sequence have been analyzed by multiple sequence analysis using Clustal matrix, the results are shown in Figure 1. It was found that the KARI sequence of Aspergillus shows the conserved patches with template between 14-280 and 421-556 amino acid residues. The conserved sequences were subjected for the prediction of their functional properties. It was found to be the sequence from 14-280 belong with NADB_Rossmann protein superfamily (Rossmann-fold NAD(P)H/NAD(P)(+) binding (NADB) domain). The NADB domain is found in numerous dehydrogenases of metabolic pathways such as glycolysis, and many other redox enzymes. NAD binding involves numerous hydrogen-bonds and van der Waals contacts, in particular H-bonding of residues in a turn between the first strand and the subsequent helix of the Rossmann-fold topology. Characteristically, this turn exhibits a consensus binding pattern similar to GXGXXG, in which the first 2 glycines participate in NAD(P)-binding, and the third facilitates close packing of the helix to the beta-strand. Typically, proteins in this family contain a second domain in addition to the NADB domain, which is responsible for specifically binding a substrate and catalyzing a particular enzymatic reaction. amino acid residues between 421-556 was found to be conserved domain of IlvC superfamily enzymes. This domain is mainly associated with, catalytic domain, involved in catalysis of acetohydroxy acids to dihydroxy valerates conversion. This reaction is the second in the synthetic pathway of the essential branched side chain amino acids valine and isoleucine.

The homology based model was generated with an objective to predict structure from its sequence with an accuracy that is comparable to the best results achieved experimentally. This, allow us to safely use rapidly generated *Insilico *protein models in all the contexts where only experimentally generated structures provide a solid basis for structure-based drug design or rational drug designing. The structure of a protein is uniquely determined by its amino acid sequence. Knowing the sequence should, at least in theory, suffice to obtain the structure. During evolution, the structure is more stable and changes much slower than the associated sequence, so that similar sequences adopt practically identical structures and distantly related sequences still fold into similar structures [33,34].

### Procheck validation

**Figure 2:**
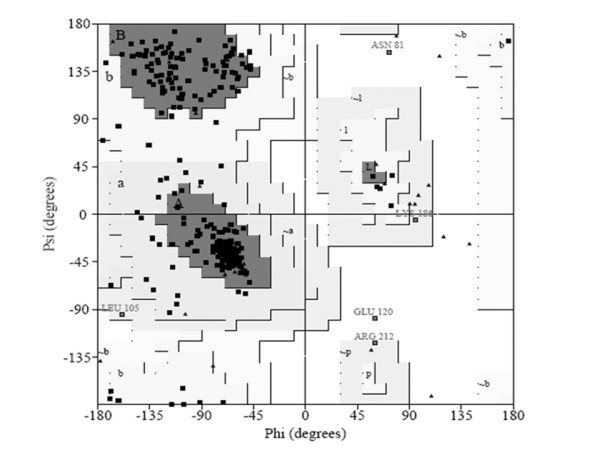
**Ramachandran plot generated by UCLA server for validation of modeled KARI**.

**Figure 3:**
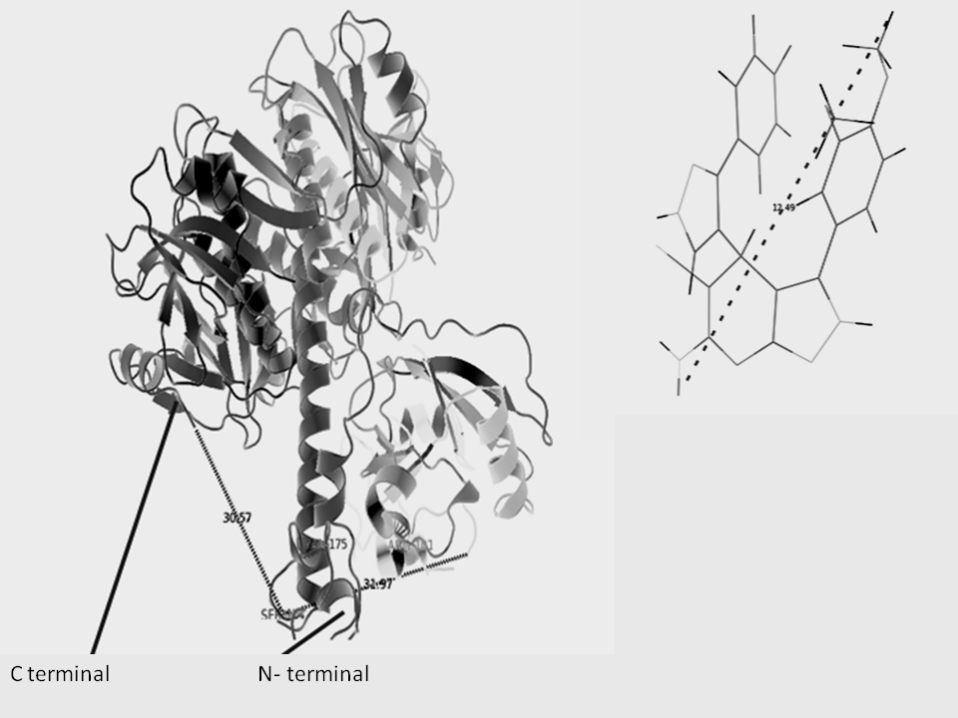
**Ribbon representation of KARI model (A) with the ligand in green color (B)**. Important residues are shown (Ser, Arg and Val.) and the distance was calculated from the active site that is serine residue (ser-184) to N-terminal (31.97Å) and C- (30.57 Å) terminals of the model receptor as calculated by pymol are shown. The inset shows the length (12.49 Å) of (4R)-6-amino-3-(3,4-dimethoxyphenyl)-4-(5-(4-fluorophenyl)-1H-pyrazol-4-yl)-2,4 dihydropyrano(2,3-c)pyrazole-5-carbonitrile.

**Figure 4:**
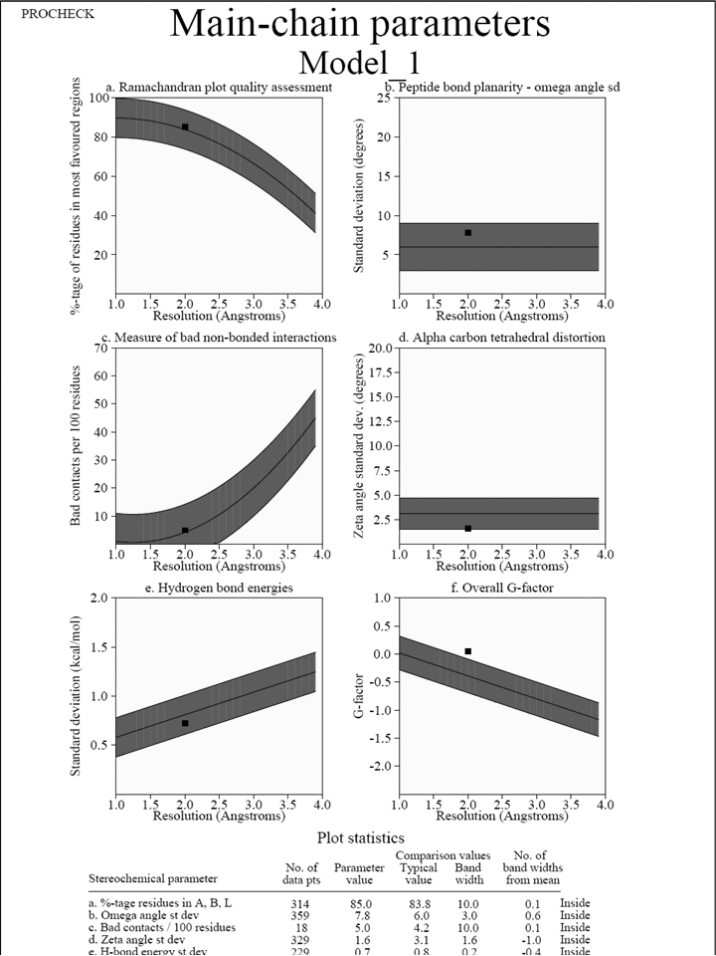
**Main chain parameter generated by WHAT IF**.

The 3D structural model of KARI gerenated by homology based model has been examined by their stereo-chemical quality, by Procheck. The phi/psi angles of 85.0% residues fell in the most favored regions, 13.4% residues lied in the additional allowed regions and 1% fell in the generously allowed regions; only 0.6% of residues lied in the disallowed conformations (Figure 2). Thus, statistical analysis suggests that the backbone conformation of our predicted model of KARI was almost as good as that of the template; the 3D conformation of the predicted model of KARI has been shown in Figure 3. In the Figure 4 main chain parameters are given. These graphs represent a comparison between the structures of the model with reference, at the similar resolution. Figure 3 and four shows various properties namely Ramachandran plot, peptide bond planarity, bad non bonded interaction's alpha tetrahedral distortion, main chain hydrogen bond energy and the overall G- factor. The overall G - factor is the measure of the overall normality of the structure. After that, residue which was present in the active site of the model found out manually and also with the help of molegro software mainly three residue of amino acid was found to be associated an active site of the model of KARI these are Arg.101-ser-184 and Val- 175. Figure 3b shows the distance from active site residue to N- terminal and C- terminal [34,35]. The residues involved in the active site as predicted by LIGSITEcsc and CASTp were Arg 101, lys 169, glu 233, Asp 223, Glu 269, ser 184 and val 175 are involved in formation of cavity for binding of ligands. A previous study on Spinach, E. coli and P. aeruginosa have shown a different active site than the prediction KARI from Aspergillus [36]. The proscane analysis for pattern elucidation was done according to Bairoch and coworkers [37]. Four patterns were found on the sequence of K.A.R.I. these patterns represent N-glycosylation site, Protein kinase C phosphorylation site, Casein kinase II phosphorylation site and N-myristoylation site. The above parametric comparison shows that the modeled structure is good for the further analysis like docking, to find some potential inhibitor.

### Docking

The sequence of KARI was submitted to drug data bank for assessment of drug like molecule, there are three molecules available with ID DB03387; DB03675; DB04497 [38,39]. Based on above information the ligand library was generated using ZINC server. This library was used for docking on KARI, using Molegro virtual docker. Six ligand molecules were selected based on their docking score. After docking, total 4475 poses were obtained. On the basis docks core, minimum energy calculation, best fit poses in the cavity. The best posse from the data was selected. The various properties and molecular structure studied ligands were mentioned in table 1. The energy score and other properties of the ligands can be selected as an inhibitor of KARI for further analysis [40].

**Table 1:** Showing various properties of ligand molecules having better score value.

	**ZINC I.D**.	Name (IUPC)	MW	Formula	Dock score
1	ZINC00720614	(4R)-6-amino-3-(3, 4-dimethoxyphenyl)-4-[5-(4-fluorophenyl)-1H-pyrazol-4-yl]-2,4-dihydropyrano[2,3c]pyrazole-5-carbonitrile	458.44	C_24_H_19_FN_6_O_3_	184.335

2	ZINC01068126	6-amino-3-(2,5-dimethoxyphenyl)-4-[5-(4-fluorophenyl)-1H-pyrazol-4-yl]-2, 4-dihydropyrano[2,3-c]pyrazole-5-carbonitrile	458.44	C_24_H_19_FN_6_O_3_	164.943

3	ZINC09291743	6-amino-4-(4-hydroxyphenyl)-3-(4-phenylmethoxyphenyl)-2,4-dihydropyrano[2,3-c]pyrazole-5-carbonitrile	436.46	C_26_H_20_N_4_O_3_	161.478

4	ZINC02090678	1-carbazol-9-yl-3-[2-(1-hydroxyethyl)benzimidazol-1-yl]propan-2-ol	387.47	C_24_H_23_N_3_O_2_	161.176

5	ZINC00663057	2-[2-(4-amino-1,2,5-oxadiazol-3-yl) benzimidazol-1-yl]-N-[(4-methylphenyl) methylideneamino]acetamide	376.39	C_19_H_17_N_7_O_2_	160.238

6	ZINC02284065	4-[5-(4-amino-1,2,5-oxadiazol-3-yl)-2-butyl-1,2,4-triazol-3-yl]-1,2, 5-oxadiazol-3-amine	291.26	C_10_H_13_N_9_O_2_	158.524

### Pharmacophore mapping

**Figure 5:**
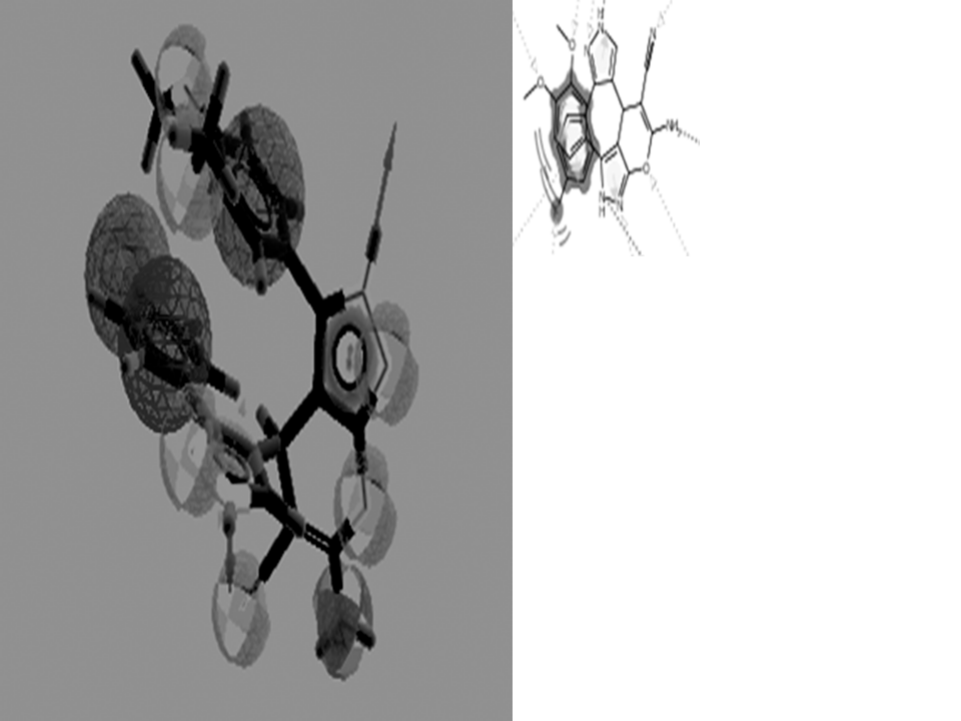
**Shows the generated pharmacophore For ZINC 00720614 with 11 active site, (A)**. Alignment of three pharmacophre was (ZINC00720614, ZINC01068126 and ZINC09291743 done with ligand scout remaining are not able to align (B).

Pharmacophore mapping was accomplished by the Ligand scout software [28]. The pharmacophore models produced were evaluated qualitatively through visual inspection and according to their ability to generate the target pharmacophores. The pharmacophore expresses constraints on the 3D structure of the molecule by specifying relative atom positions that should be maintained to increase the likelihood that the molecule will bind with the receptor site [41,42]. For all six ligand pharmacophore was generated. Figure 5 shows pharmacophore model generated with ZINC00720614, which is found to be better and could be use as a skeleton for design new class of drugs. The other Ligands namely ZINC01068126, ZINC09291743, ZINC02284065, ZINC00663057, ZINC02090678 was also used to generate pharmacophore models for comparative analysis [23,24,43].

### ADME/Tox properties

Absorption, Distribution, Metabolism, Excretion and Toxicity (ADME/Tox) are main five parameters to test the drug likeness of a molecule. ADME/Tox was tested by the pharma algorithm [44]. The table 2, summaries above-mentioned properties were given. Thus, the pharma algorithm gives an idea about drug likeness of the ligand molecule by studying this (table 2) can be able to know the oral bioavailability, absorption and the toxic effect of drug like molecule. By this study, it becomes easy to optimize the lethal doses of any molecule without killing any animal, which reduces the cost [45]. Oral bioavailability of drug must be low, and shows the oral bioavailability of all six ligands (Table 2). The analysis of the World Drug Index (WDI), which lead to Lipinski's 'rule-of-five' identifies several critical properties that should be considered for compounds with oral delivery in mind. These properties, which are usually viewed more as guidelines rather than absolute cutoffs, are molecular mass < 500 daltons (Da), calculated octanol/water partition coefficient (CLOGP) < 5, number of hydrogen-bond donors < 5 and number of hydrogen-bond acceptors < 10. Thus, such studies point the most important physicochemical properties and structural characteristic of a good drug in the context of our current knowledge. These properties are then typically used to construct predictive ADME models and create the basis for what has been called property-based design [46]. The comparative ADME/Tox analysis of these Ligands encouraging them to use as drug like molecule, as accord [23,24,43,46,47].

**Table 2:** Pharmacophore properties of best possible drug like molecules

**ZINCI.D**.	Predicted values- oral Bioavailability	Predicted Values - Passive Absorption (Human Intestinal)	Predicted Values - Probabilities of Health Effects	Predicted Values- acute toxicity (LD50, Mouse)				
				
					LD50 (mg/kg)	pLD50	Lower limit	Upper limit
ZINC00720614	less than 30%	**Maximum passive absorption: 100%** Contribution from: Trancellular route = 100% Paracellular route = 0% **Permeability:** Human Jejunum scale (pH = 6.5): Pe, Jejunum = 3.09 × 10^-4 ^cm/s **Absorption rate:** K_a _= 0.091 min^-1^	Blood- 0.99 Cardiovascular system-1.00 Gastrointestinal system-1.00 Kidney -0.99 Liver- 0.98 Lungs-0.82	**Ip**	1000.0	-0.34	-1.17	0.42
				
				**O**	800.0	-0.24	-1.81	0.92
				
				**Iv**	62.0	0.87	-0.18	2.24
				
				**S**	950.0	-0.32	-1.90	1.43

ZINC01068126	less than 30%	**Ma × imum passive absorption: 100%** Contribution from: Trancellular route = 100% Paracellular route = 0% **Permeability:** Human Jejunum scale (pH = 6.5): Pe, Jejunum = 3.42 × 10^-4 ^cm/s **Absorption rate**: K_a _= 0.093 min^-1^	Blood- 0.99 Cardiovascular system-1.00 Gastrointestinal system-1.00 Kidney -0.99 Liver- 0.98 Lungs-0.82	**Ip**	620.0	-0.13	-1.01	0.64
				
				**O**	610.0	-0.13	-1.69	1.04
				
				**Iv**	48.0	0.98	-0.05	2.27
				
				**S**	640.0	-0.15	-1.73	1.56

ZINC09291743	less than 30%	**Maximum passive absorption: 100%** Contribution from: Trancellular route = 100% Paracellular route = 0% **Permeability:** Human Jejunum scale (pH = 6.5): Pe, Jejunum = 5.94 × 10^-4 ^cm/s **Absorption rate**: K_a _= 0.100 min^-1^	Blood- 0.98 Cardiovascular system-.96 Gastrointestinal system-1.00 Kidney -0.96 Liver- 0.92 Lungs-0.97	**Ip**	590.0	-0.13	-1.13	0.73
				
				**O**	850.0	-0.29	-1.76	0.36
				
				**Iv**	43.0	1.00	0.20	2.40
				
				**S**	920.0	-0.32	-1.91	1.42

ZINC02090678	between 30% and 70%	**Maximum passive absorption: 100%** Contribution from: Trancellular route = 100% Paracellular route = 0% **Permeability:** Human Jejunum scale (pH = 6.5): Pe, Jejunum = 5.42 × 10^-4 ^cm/s **Absorption rate:** K_a _= 0.100 min^-1^	Blood- 0.88 Cardiovascular system-0.82 Gastrointestinal system-0.82 Kidney -0.31 Liver- 0.63 Lungs-0.98	**Ip**	420.0	-0.04	-1.08	0.85
				
				**O**	1100.0	-0.45	-1.97	0.33
				
				**Iv**	54.0	0.85	-0.28	2.30
				
				**S**	590.0	-0.19	-1.83	1.32

ZINC00663057	between 30% and 70%	**Maximum passive absorption: 100%** Contribution from: Trancellular route = 100% Paracellular route = 0% **Permeability:** Human Jejunum scale (pH = 6.5): Pe, Jejunum = 3.09 × 10^-4 ^cm/s **Absorption rate:** K_a _= 0.091 min^-1^	Blood- 0.61 Cardiovascular system-0.36 Gastrointestinal system-0.53 Kidney -0.55 Liver- 0.42 Lungs-0.80	**Ip**	300.0	0.09	-0.73	0.71
				
				**O**	1000.0	-0.43	-1.89	0.06
				
				**Iv**	84.0	0.65	-0.31	1.84
				
				**S**	440.0	-0.07	-1.86	1.07

ZINC02284065	between 30% and 70%	**Maximum passive absorption: 100%** Contribution from: Trancellular route = 99% Paracellular route = 1% **Permeability:** Human Jejunum scale (pH = 6.5): Pe, Jejunum = 1.07 × 10^-4 ^cm/s **Absorption rate:** K_a _= 0.034 min^-1^	Blood- 0.77 Cardiovascular system-0.01 Gastrointestinal system-0.77 Kidney -0.38 Liver- 0.43 Lungs-0.22	**Ip**	120.0	0.40	-0.73	1.66
				
				**O**	510.0	-0.24	-1.63	0.66
				
				**Iv**	110.0	0.42	-1.02	2.16
				
				**S**	570.0	-0.29	-1.86	1.75

** Ip- Intraperitoneal; O - Oral; Iv- Intravenous; S-Subcutaneous

## Conclusion

Our previous work in which we have analyzed the metabolic pathways in the finding of essential protein, which could be targeted for drug designing. Comparative study of metabolome of the Aspergilli bestows the idea that essential enzymes can be targeted for antifungal drug designing [8], and 40 imperative proteins were identified from Aspergillus. Out of these putative targets, KARI was selected for present work, as it was found to be non-homologous protein in comparison with human protein. Therefore, targeting this protein will be Safe. Since 3D structure of KARI from Aspergilli was not reported yet so a model of this enzyme was produced by Swiss model. That model was validated by procheck and WHAT IF, programs. The structure of KARI was modeled Insilico based on X- ray crystallography structure of KARI B- chain of rice was used as the template. The ligand library was generated with the help of the drug bank from the zinc database. About 495, ligands were used in the preparation of the ligand library for docking. As a result, six ligands ZINC00720614, ZINC01068126, ZINC09291743, ZINC02090678, ZINC006637 and ZINC02284065 was selected based on docking score. It was evaluated that serine-184 was found to be a key residue along with valine and Arganine residue to form a binding site. These findings advance our knowledge on specific interactions on ZINC00720614, ZINC01068126, ZINC09291743, ZINC02090678, ZINC006637 and ZINC02284065 bind with KARI-receptor. Pharmacophore analysis was suggested about the active site of drug like molecule, and 11 such sites were deduced on ZINC00720614 ligand. This number of the active sites showed that ZINC00720614 is the best ligand molecule among all selected ligands. Maximum number of active site in a ligand molecule shows the highest chances of binding and also of lowest binding energy. The bioavailability, absorption and toxicity of the drug-like molecule were studied by the pharma algorithm. Oral bioavailability stands for the fraction of drug available for the mouth this six ligand molecule can be the potential drug for Aspergillosis. Uniformity of absorption of a drug-like molecule is important factors when considering its formulation and relies upon system. The minimum absorption rate constant ka value of 0.17 to 0.32 per hour necessary for about 80-95% absorption over 9-12 hrs. Absorption rate of drug provides an idea about the rate of absorption of drug like molecule and the absorption rate of these should be high, so that these molecules must be available for biological system. Thus on the basis information obtained from ADMET properties study time and cost both can be saved along with life of various animals. Therefore, homology based rational drug designing can be a successful approach for designing of potent antifungal drug. It still needed to explore some more invivo experimentation for complete evaluation as a drug. Using this selectable approach for designing the drug, a researcher can minimize the try and hit methodology, thus can save the time, cost and life of test animals. We found KARI as a potential target while design the drug against Aspergillus.

## Competing interests

The authors declare that they have no competing interests.

## Authors' contributions

VKM has designed the experimental; SK has done the computational and software operation work along VKM. VKM and EKK have analyzed the resulted. The manuscript was written by VKM and EKK. All authors read and approved the final manuscript.
